# Identification of bis-benzylisoquinoline alkaloids as SARS-CoV-2 entry inhibitors from a library of natural products

**DOI:** 10.1038/s41392-021-00531-5

**Published:** 2021-03-23

**Authors:** Chang-Long He, Lu-Yi Huang, Kai Wang, Chen-Jian Gu, Jie Hu, Gui-Ji Zhang, Wei Xu, You-Hua Xie, Ni Tang, Ai-Long Huang

**Affiliations:** 1 Key Laboratory of Molecular Biology for Infectious Diseases (Ministry of Education), Institute for Viral Hepatitis, Department of Infectious Diseases, The Second Affiliated Hospital, Chongqing Medical University, Chongqing, China; 2 Key Laboratory of Medical Molecular Virology (MOE/NHC/CAMS), Department of Medical Microbiology and Parasitology, School of Basic Medical Sciences, Shanghai Medical College, Fudan University, Shanghai, China; 3 Children’s Hospital, Fudan University, Shanghai, China

**Keywords:** Microbiology, Drug development

**Dear Editor,**

Coronavirus disease 2019 (COVID-19) caused by severe acute respiratory syndrome coronavirus 2 (SARS-CoV-2) is a major public health issue. The spike (S) protein mutation D614G became dominant in SARS-CoV-2 during a global pandemic, which displayed increased infectivity.^1^ Entry of a virus into host cells is one of the most critical steps in the viral life cycle. Since blockade of the entry process is a promising therapeutic option for COVID-19, research attention has been focused on the discovery of viral entry inhibitors. Although SARS-CoV-2 entry inhibitor development is very attractive, no candidates have progressed into clinical trials yet.

Using a luciferase-expressing pseudovirus encoding SARS-CoV-2 S (G614) protein, a library of 188 natural compounds (Supplementary Tab. S1) was screened in 293T-ACE2 cells (HEK 293T cells overexpressing human angiotensin-converting enzyme 2) to find novel anti-SARS-CoV-2 entry inhibitors. Vesicular stomatitis virus G (VSV-G) pseudovirus was used as a control to exclude compounds targeting the lentiviral backbone. A workflow chart of screening is shown in Fig. 1a. After a preliminary screening, 41 compounds associated with a relative infection rate <30% (Fig. 1b) were identified. We selected 19 compounds with low cytotoxicity for further testing (Supplementary Tab. S2, Fig. S1). Among the 19 hits, nine compounds (SC9, SC161, SC171, SC182–187) with relatively high activity (EC_50_ < 10 μM), low cytotoxicity (CC_50_ > 20 μM), and high specificity (SI > 10, VSV-G EC_50_ > 20 μM) were selected for subsequent analyses. Notably, all these compounds were bis-benzylisoquinoline alkaloids except SC171.

**Fig. 1:**
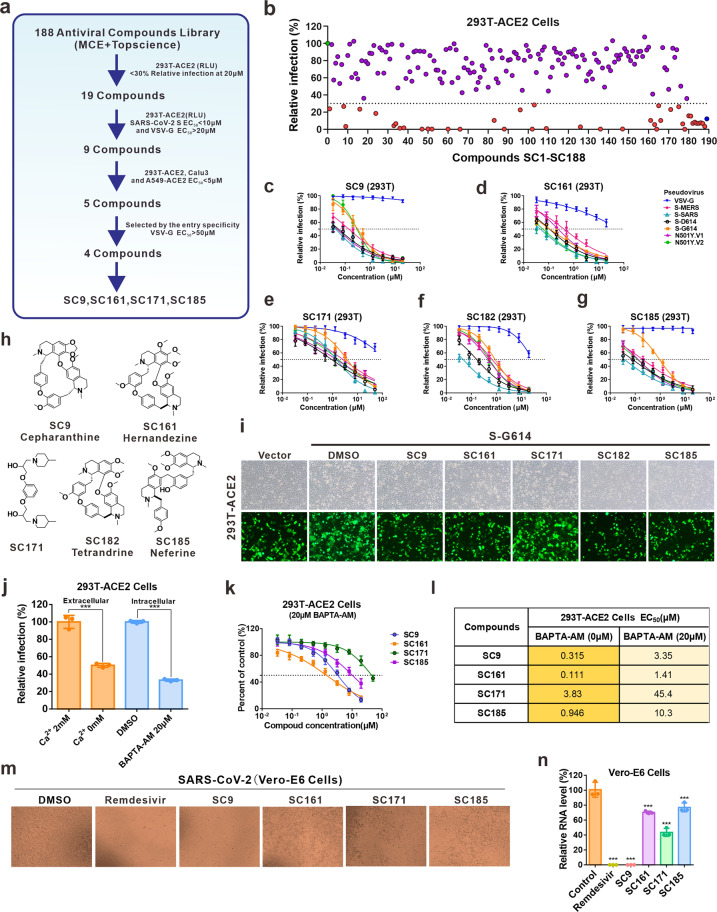
Identification of bis-benzylisoquinoline alkaloids as SARS-CoV-2 entry inhibitors. **a** Schematic diagram of the screening workflow with selection criteria for hits outlined. **b** Scatter plot of primary screening of 188 compounds against S-G614 infection. Inhibition ratios for all drugs obtained in a preliminary screening are represented by scattered points. Red dots indicate the 41 compounds with an inhibition rate ≥70%. DMSO (green dot) and aloxistatin (blue dot) were used as a negative and positive control, respectively. **c**–**g** Dose-response curves of five selected compounds (**c**) SC9, (**d**) SC161, (**e**) SC171, (**f**) SC182, (**g**) SC185 on VSV-G, S-D614, S-G614, S-SARS, S-MERS, N501Y.V1, and N501Y.V2 pseudoviruses. **h** Chemical structures of SC9, SC161, SC171, SC182, and SC185. **i** Inhibitory effect of SC9, SC161, SC171, SC182, and SC185 at 5 μM on SARS-CoV-2 S mediated cell-cell fusion. **j** Effect of extracellular and intracellular Ca^2+^ depletion on S-G614 pseudovirus entry in 293T-ACE2 cells. **k**–**l** Inhibition curves (**k**) and EC_50_ values (**l**) of the compounds against S-G614 pseudovirus entry in the presence of 20 μM BAPTA-AM. **m** The inhibitory effect of the compounds on native SARS-CoV-2 infection by observing their cytopathogenic effects. SC9, SC161, SC171, and SC185 were tested at 10 μM, and DMSO and remdesivir (5 μM) were used as a negative and positive control, respectively. **n** The relative viral RNA levels in the SC9, SC161, SC171, and SC185 (10 μM) treatment groups were 0.08%, 70.27%, 43.55%, and 76.98% respectively. *P < 0.05; **P < 0.01; ***P < 0.001. All experiments were repeated at least three times

Next, we analyzed the relationship between the antiviral efficacy of the nine selected compounds against S-G614 pseudovirus and the timing of treatment (Supplementary Fig. S2). We divided the pseudovirus-based entry assay into three stages: pretreatment (pre-entry), viral entry, and post-entry stage. In total, eight experimental groups were set up for each compound, including seven treatment groups (A–G) and a control group. Importantly, pretreatment with each compound (group B) significantly inhibited S-G614 pseudovirus infection. In the viral entry stage (group C), the compounds exerted similar suppressive effects. However, in the post-entry stage (group D), none of the compounds showed any inhibitory effect. These data demonstrated that the nine selected compounds showed high blockade efficacy presenting in both pre-entry and entry steps, indicating that they target host factors during viral infection.

Cell lines mimicking important aspects of respiratory epithelial cells should be used when analyzing the anti-SARS-CoV-2 activity. Hence, we determined their EC_50_ values against S-G614 pseudovirus in Calu-3 and A549 cells (Supplementary Fig. S3a–i). Five compounds (SC9, SC161, SC171, SC182, and SC185) with EC_50_ < 10 μM in all three cell lines were selected for subsequent experiments.

To determine whether these compounds have broad-spectrum antiviral effects against other betacoronaviruses as well as recently emerged SARS-CoV-2 variants, we constructed S-D614, N501Y.V1 (B.1.1.7), N501Y.V2 (B.1.351), S-SARS, and S-MERS pseudoviruses using the same lentiviral system as S-G614, and then determined the EC_50_ values of SC9 (cepharanthine, Fig. 1c), SC161 (hernandezine, Fig. 1d), SC171 (Fig. 1e), SC182 (tetrandrine, Fig. 1f), and SC185 (neferine, Fig. 1g) against these pseudoviruses in 293T cells expressing ACE2 or dipeptidyl peptidase 4 (DPP4) (Fig. 1h). Interestingly, SC9, SC161, SC171, and SC185 exhibited highly potent pan-inhibitory activity against S-pseudotyped coronaviruses including two emerging SARS-CoV-2 variants N501Y.V1 and N501Y.V2, reported in the United Kingdom and South Africa (Supplementary Fig. S3j). As SARS-CoV and SARS-CoV-2 have been reported to enter host cells via binding to ACE2, and while DPP4 is critical for MERS-CoV entry, it could be ruled out that these five compounds interfere with ACE2 to block pseudovirus entry.

Then, we used competitive ELISAs and thermal shift assays to determine whether these five compounds interact with the receptor-binding domain (RBD) in the S protein of SARS-CoV-2. SBP1, a peptide derived from the ACE2 α1 helix, bound RBD of SARS-CoV-2 and exhibited a weak ability to inhibit the entry of S-G614 pseudovirus (Supplementary Fig. S4a), whereas the interaction between SC9, SC161, SC171, or SC185 and RBD was negligible (Supplementary Fig. S4b–d). Thus, the blockade of virus entry by these candidate compounds is not related to the interaction with RBD of SARS-CoV-2.

Following attachment to the host receptor, the membrane fusion process mediated by the S protein of SARS-CoV-2 plays an important role in viral entry. Our data indicated that the above five compounds may target host cells to inhibit coronavirus entry. Therefore, we examined whether these compounds perturb SARS-CoV-2 induced cell fusion. Cell-cell fusion assay exhibited that SC9, SC161, SC182, and SC185 at 5 μM potently inhibited SARS-CoV-2 S-mediated membrane fusion of 293T-ACE2 cells with approximately 90% decrease of fusion rates (Fig. 1i, Supplementary Fig. S4e). Since calcium ion (Ca^2+^) plays a critical role in SARS-CoV or MERS-CoV S-mediated membrane fusion,^2^ calcium channel blockers (CCBs), originally used to treat cardiovascular diseases, are supposed to have a high potential to treat SARS-CoV-2 infections.^3^ Consistently, calcium-free medium or intracellular Ca^2+^ chelation with BAPTA-AM significantly diminished SARS-CoV-2 pseudovirus infection (Fig. 1j, Supplementary Fig. S4f–i), suggesting that Ca^2+^ is also required for SARS-CoV-2 entry. The identified bis-benzylisoquinoline alkaloids had been reported as CCBs.^4^ Herein, bis-benzylisoquinoline alkaloids may abolish S–ACE2-mediated membrane fusion by targeting the host calcium channel. Upon pretreatment with BAPTA-AM, the bis-benzylisoquinoline CCBs had approximately 10-fold higher EC_50_ values than those without BAPTA-AM pretreatment (Fig. 1k–l, Supplementary Fig. S4j–k). Besides, perturbation of the cholesterol biosynthesis pathway with the CCB amlodipine reduced viral infection.^5^ Consistent herewith, the bis-benzylisoquinoline CCBs upregulated intracellular cholesterol level (Supplementary Fig. S4l), which also likely contributed to the inhibition of viral infection. These data indicated that blockade of S-G614 pseudovirus entry by bis-benzylisoquinoline CCBs mainly depends on calcium homeostasis.

Finally, the antiviral activities of SC9 (cepharanthine), SC161 (hernandezine), SC171, and SC185 (neferine) were confirmed in Vero E6 cells infected with native SARS-CoV-2. Virus-induced cytopathogenic effect and the viral RNA levels were partially inhibited by these compounds, with SC9 (cepharanthine) at the highest efficacy (Fig. 1m–n). The results showed that these compounds inhibited SARS-CoV-2 to varying degrees and may be useful as leads for SARS-CoV-2 therapeutic drug development.

In summary, we reported a set of bis-benzylisoquinoline alkaloids as pan-coronavirus entry inhibitors. These host-targeted inhibitors effectively protected different cell lines (293T-ACE2, Calu-3, and A549) from infection by different coronaviruses (SARS-CoV, MERS-CoV, SARS-CoV-2 [S-D614, S-G614, and N501Y variants]) in vitro. The compounds blocked host calcium channels, thus inhibiting Ca^2+^-mediated fusion and suppressing virus entry. Considering the effectiveness of CCBs in the control of hypertension, our study provided clues to support that CCBs may be helpful for treating coronavirus infection in patients with hypertension.

## Supplementary information

Supplementary Materials
