# Supplementary material for: Identification of bis-benzylisoquinoline alkaloids as SARS-CoV-2 entry inhibitors from a library of natural products
Source: Signal Transduct Target Ther. 2021 Mar 23;6:131. doi: 10.1038/s41392-021-00531-5 (PMC7985570; doi:10.1038/s41392-021-00531-5)
Supplement: Supplementary file 1 — Supplementary Materials [file 41392_2021_531_MOESM1_ESM.docx]

Supplementary Materials for

**Identification of bis-benzylisoquinoline alkaloids as SARS-CoV-2 entry inhibitors from a library of natural products**

Chang-Long He^1,^ ^#^, Lu-Yi Huang^1,^ ^#^, Kai Wang^1,^ ^#^, Chen-Jian Gu^2^, Jie Hu^1^, Gui-Ji Zhang^1^, Wei Xu^2^, You-Hua Xie^2, 3,^*, Ni Tang^1,^*, Ai-Long Huang^1,^*

Correspondence to: {[ahuang@cqmu.edu.cn](mailto:ahuang@cqmu.edu.cn), [nitang@cqmu.edu.cn](mailto:nitang@cqmu.edu.cn), [yhxie@fudan.edu.cn](mailto:yhxie@fudan.edu.cn)}shibojiang@fudan.edu.cn；lul@fudan.edu.cn；zhyuan@shmu.edu.cn

**This PDF file includes:**

Figure S1 to S4

Table S1 to S2

Materials and methods

**Figure S1**


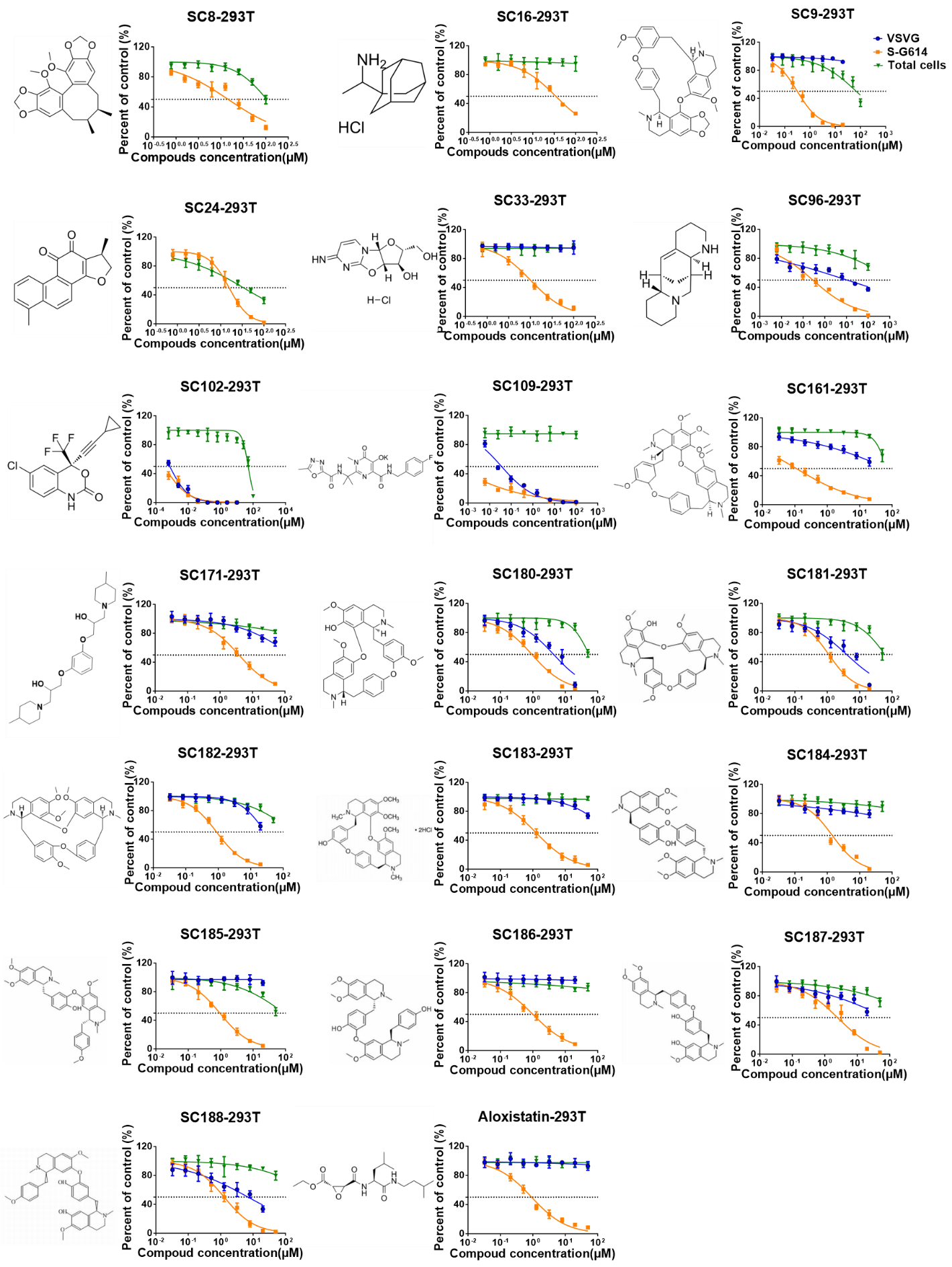


**Fig. S1.** The chemical structures and inhibitory activities of 19 hits and aloxistatin against SARS-CoV-2 S-G614, VSV-G pseudoviruses and cell viability assay of 293T-ACE2.

**Figure S2**


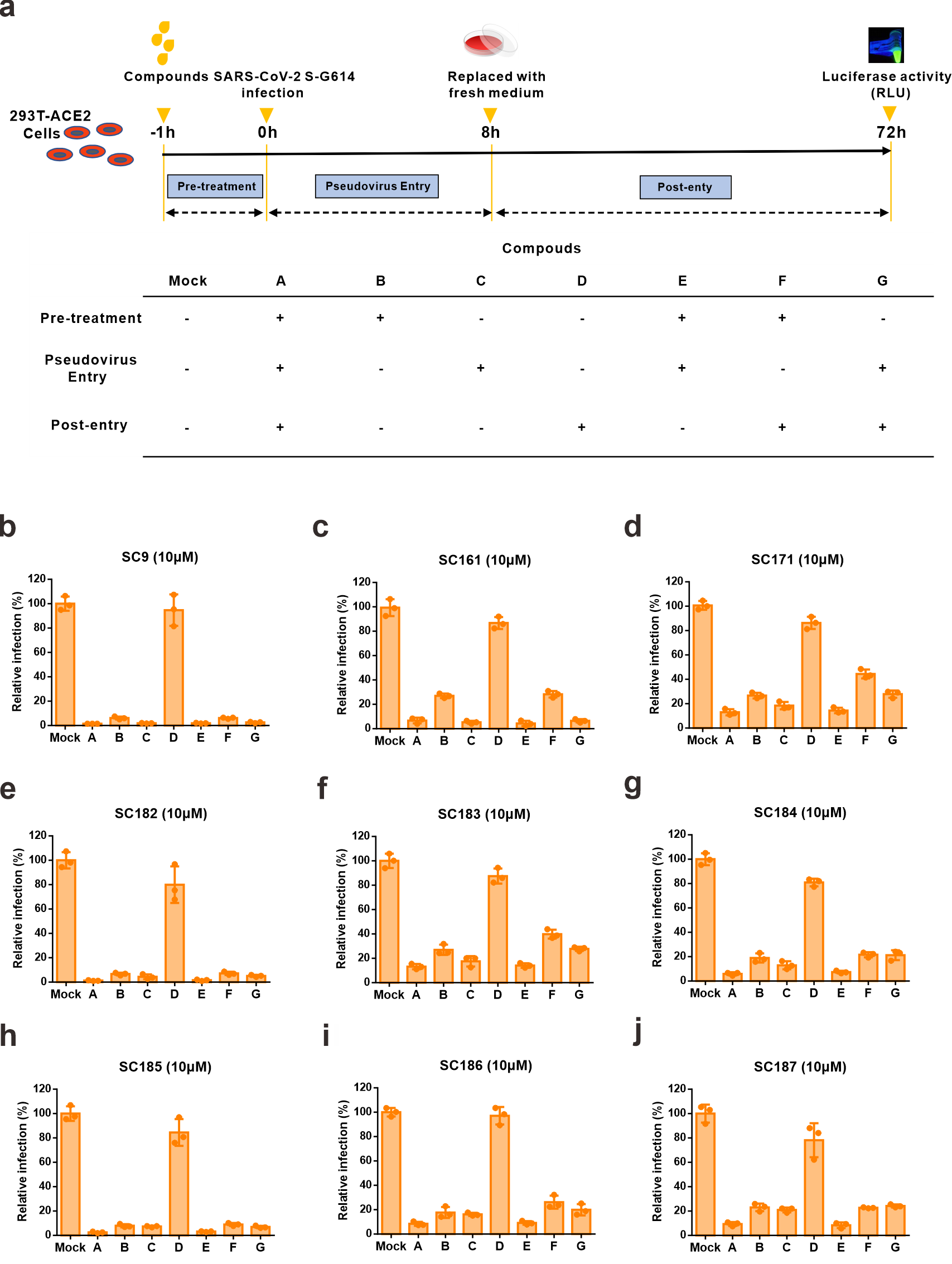


**Fig. S2** Effect of treatment timing on the efficacy of nine selected compounds on SARS-CoV-2 S-G614 pseudovirus entry. **a** Treatment timing diagrams for the nine selected compounds. HEK 293T cells were treated with the nine compounds or DMSO before, during, or after S-G614 pseudovirus entry. Seven treatment conditions (A–G) were tested for each compound. **b–j** Inhibitory effects of **(b)** SC9, **(c)** SC161, **(d)** SC171, **(e)** SC182, **(f)** SC183, **(g)** SC184, **(h)** SC185, **(i)** SC186, and **(j)** SC187 on the entry of S-G614 pseudovirus at different treatment time points. All experiments were repeated at least three times.

**Figure S3**


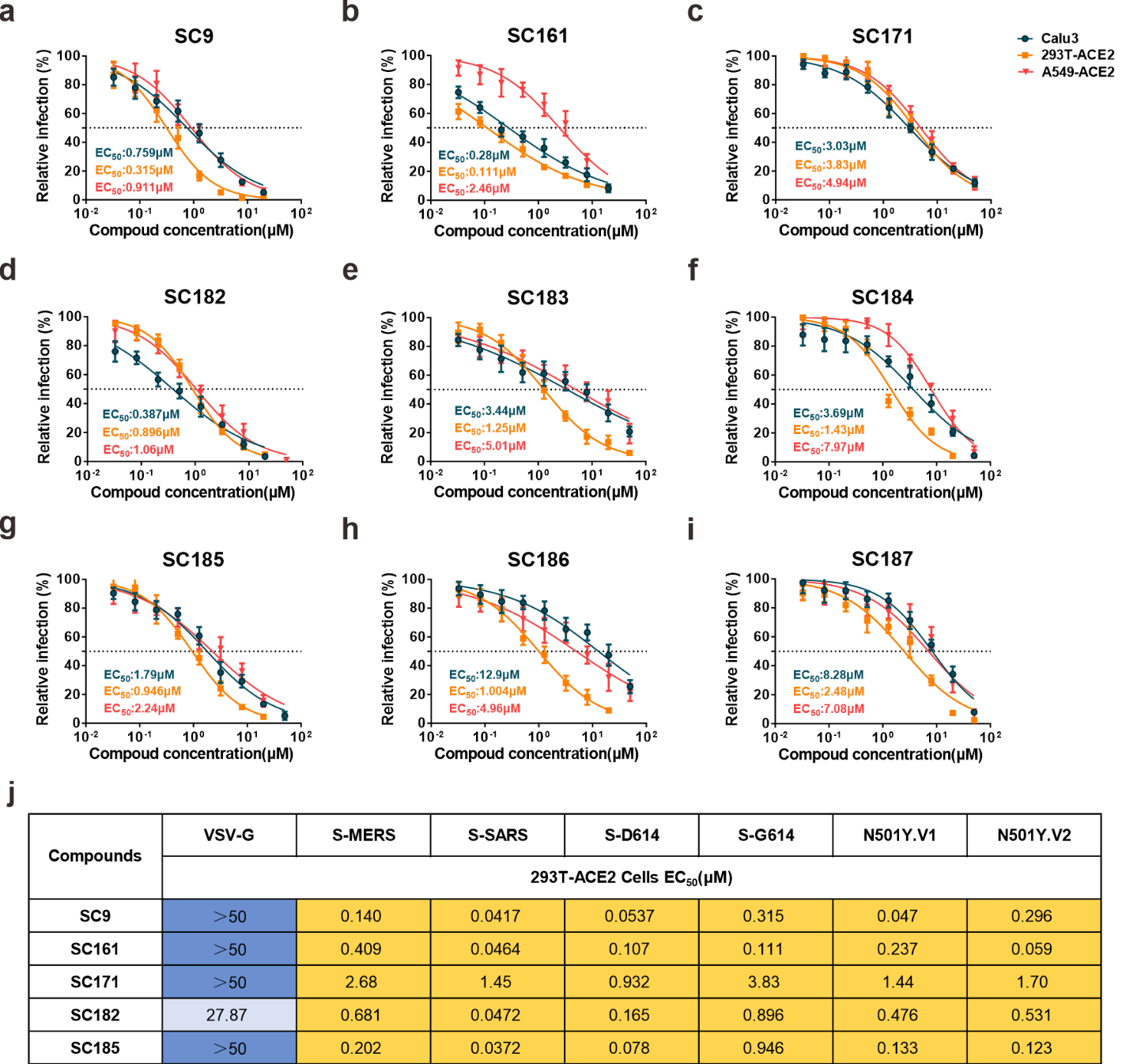


**Fig. S3** Efficacy of compounds in Calu3, 293T-ACE2, and A549 cell lines against different coronaviruses. **a-i** Inhibitory effects of **(a)** SC9, **(b)** SC161, **(c)** SC171, **(d)** SC182, **(e)** SC183, **(f)** SC184, **(g)** SC185, **(h)** SC186, and **(i)** SC187 against S-G614 infection in the three cell lines. All experiments were repeated at least three times. (**j**) EC_50_ values of five selected compounds against entry of different pseudoviruses. All experiments were repeated at least three times.

**Figure S4**


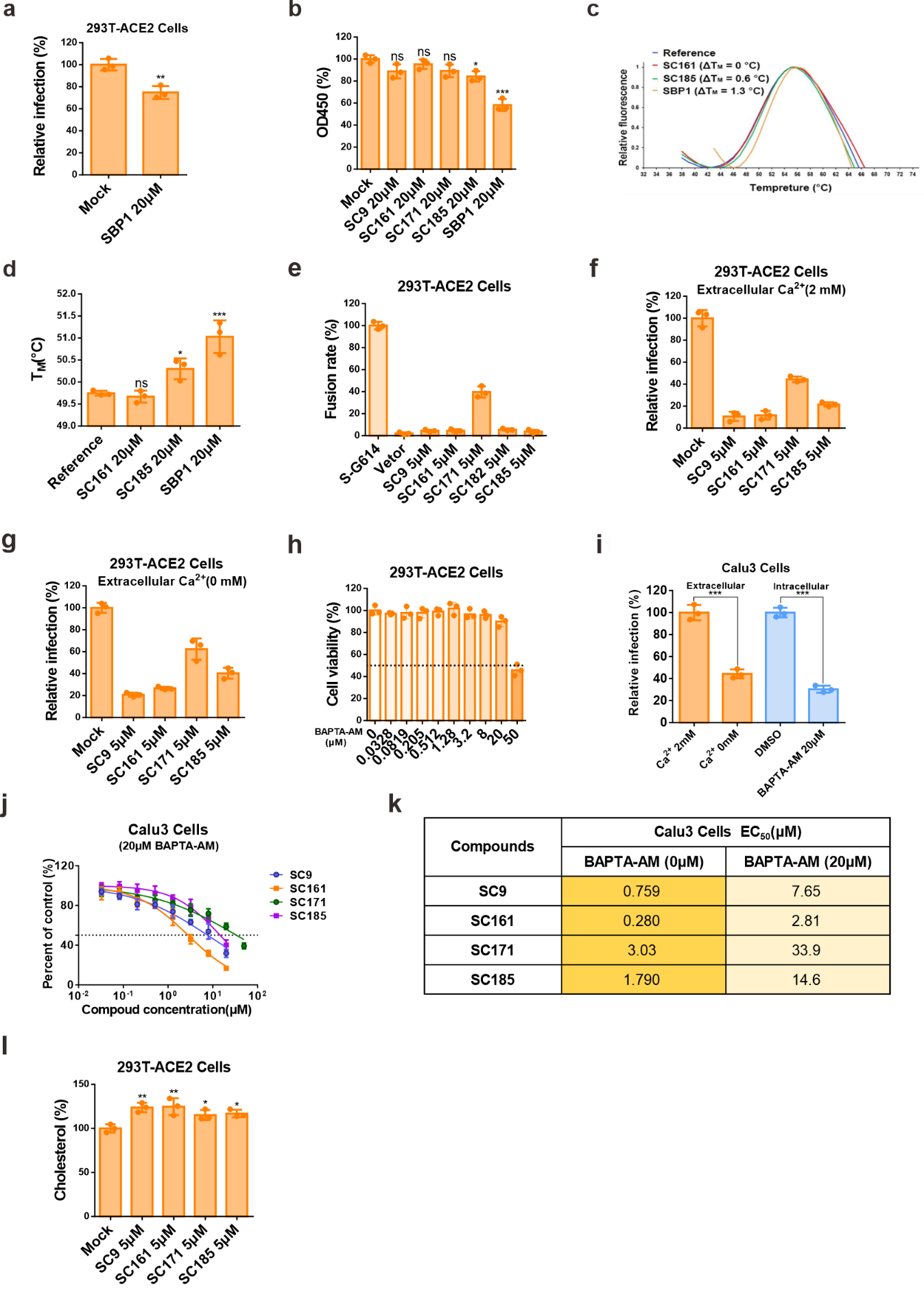


**Fig. S4** Investigation of the mechanism of the selected compounds. **a** Inhibitory effect of the ACE2 peptide SBP1 at 20 μM on the invasion of S-G614 pseudovirus. **b** Binding of pre-coated His-RBD with compounds at 20 μM as determined by competitive ELISA. SBP1 and DMSO were used as a positive and negative control, respectively. **c** Melting curves for His-RBD (10 μM) incubated with 20 μM of each compound tested by differential scanning fluorimetry. **d** T_M_ values of His-RBD incubated with SC161, SC185, or SBP1. **e** Cell–cell fusion rate in the presence of 5 μM SC9, SC161, SC171, SC182, or SC185. The fusion rate in a DMSO-treated group was set as 100%. **f** Effect of 5 μM of each compound on S-G614 pseudovirus entry into 293T-ACE2 cells in medium supplemented with 2 mM CaCl_2_. **g** Effect of 5 μM of each compound on S-G614 pseudovirus entry in calcium-depleted culture medium. **h** Cell viability assay of 293T-ACE2 cells treated with increasing concentrations of BAPTA-AM (0–50 μM). **i** Effect of extracellular and intracellular Ca^2+^ depletion on S-G614 pseudovirus entry in Calu-3 cells. **j-k** Inhibition curves (**j**) and EC_50_ values (**k**) of the compounds against S-G614 pseudovirus entry in the presence of 20 μM BAPTA-AM. **l** Cholesterol (normalized to total protein) in 293T-ACE2 cells treated with the compounds or DMSO for 24 h. **P* < 0.05; ***P* < 0.01; ****P* < 0.001. ns, not significant. All experiments were repeated at least three times.

**Table S1**

| **Supplementary Tab. 1** List of 188 compounds. | | | | | | | | |
| --- | --- | --- | --- | --- | --- | --- | --- | --- |
| **Code** | **Compounds Name** | **M. Wt** | **Code** | **Compounds Name** | **M. Wt** | **Code** | **Compounds Name** | **M. Wt** |
| SC1 | Xanthohumol | 354.40 | SC64 | 2,2'-Anhydrouridine | 226.19 | SC127 | 3,4-Dimethoxycinnamic acid | 208.21 |
| SC2 | Lycorine | 287.31 | SC65 | Tricin | 330.29 | SC128 | Fumagillin | 458.54 |
| SC3 | Oxindole | 133.15 | SC66 | Tubercidin | 266.25 | SC129 | Geniposide | 388.37 |
| SC4 | L-Lysine | 146.19 | SC67 | (-)-Epicatechin gallate | 442.37 | SC130 | Valacyclovir (hydrochloride) | 360.80 |
| SC5 | Acyclovir | 225.20 | SC68 | Isochlorogenic acid A | 516.45 | SC131 | Guanosine | 283.24 |
| SC6 | Oxymatrine | 264.36 | SC69 | Daidzin | 416.38 | SC132 | Sophocarpine (monohydrate) | 264.36 |
| SC7 | Catalpol | 362.33 | SC70 | 2-Phenylethanol | 122.16 | SC133 | Probucol | 516.84 |
| SC8 | Schisandrin C | 384.42 | SC71 | PCL 016 | 123.11 | SC134 | Kaempferide | 300.26 |
| SC9 | Cepharanthine | 606.71 | SC72 | Pyridoxal phosphate | 247.14 | SC135 | Genkwanin | 284.26 |
| SC10 | Oleanonic acid | 454.68 | SC73 | Dipotassium glycyrrhizinate | 861.02 | SC136 | Deapioplatycodin D | 1093.21 |
| SC11 | N6-Methyladenosine | 281.27 | SC74 | Daphnoretin | 352.29 | SC137 | Acetylcysteine | 163.19 |
| SC12 | α-Vitamin E | 430.71 | SC75 | Coptisine (chloride) | 355.77 | SC138 | Chelidonine | 353.37 |
| SC13 | Gentiopicroside | 356.32 | SC76 | Methyl gallate | 184.15 | SC139 | L-Cycloserine | 102.09 |
| SC14 | Shikonin | 288.30 | SC77 | Scutellarin | 462.36 | SC140 | Coumarin | 146.14 |
| SC15 | Valpromide | 143.23 | SC78 | Picroside II | 512.46 | SC141 | 20(R)-Ginsenoside Rh2 | 622.87 |
| SC16 | Rimantadine (hydrochloride) | 215.76 | SC79 | Maslinic acid | 472.70 | SC142 | Mecarbinate | 233.26 |
| SC17 | Impulsin | 299.49 | SC80 | Schisandrin A | 416.51 | SC143 | Azulene | 128.17 |
| SC18 | Brefeldin A | 280.36 | SC81 | Glycitin | 446.40 | SC144 | 3,4'-Dihydroxyflavone | 254.24 |
| SC19 | Cynarin | 516.45 | SC82 | Amentoflavone | 538.46 | SC145 | L-Chicoric Acid | 474.37 |
| SC20 | 2-Deoxy-D-glucose | 164.16 | SC83 | Ivermectin | 875.09 | SC146 | Danthron | 240.21 |
| SC21 | (-)-α-Pinene | 136.23 | SC84 | Aucubin | 346.33 | SC147 | Doxorubicin (hydrochloride) | 579.98 |
| SC22 | Naringenin | 272.25 | SC85 | Corilagin | 634.45 | SC148 | Gomisin G | 536.57 |
| SC23 | Hinokitiol | 164.20 | SC86 | Vidarabine | 267.24 | SC149 | Pentoxifylline | 278.31 |
| SC24 | Dihydrotanshinone I | 278.30 | SC87 | Trigonelline chloride | 173.60 | SC150 | Andrographolide | 350.45 |
| SC25 | Thiamine hydrochloride | 337.27 | SC88 | Harringtonine | 531.59 | SC151 | Mycophenolic acid | 320.34 |
| SC26 | Osthole | 244.29 | SC89 | Oleanolic Acid | 456.70 | SC152 | Sennoside A | 862.74 |
| SC27 | D-Pinitol | 194.18 | SC90 | Octyl gallate | 282.33 | SC153 | Punicalin | 782.53 |
| SC28 | Angelicin | 186.16 | SC91 | Hyperoside | 464.38 | SC154 | Punicalagin | 1084.72 |
| SC29 | N-Acetylneuraminic acid | 309.27 | SC92 | Limonin | 470.51 | SC155 | Verbascoside | 624.59 |
| SC30 | Baicalin | 446.36 | SC93 | Epigoitrin | 129.18 | SC156 | Bergenin | 328.27 |
| SC31 | Ginsenoside Rb1 | 1109.29 | SC94 | Ginsenoside Rb2 | 1079.27 | SC157 | Chlorogenic acid | 354.31 |
| SC32 | Oglufanide | 333.34 | SC95 | Rutin | 610.52 | SC158 | Tunicamycin | 844.94 (n=10) |
| SC33 | Ancitabine (hydrochloride) | 261.66 | SC96 | Aloperine | 232.36 | SC159 | L-Lysine hydrochloride | 182.65 |
| SC34 | Camptothecin | 348.35 | SC97 | Pentagalloylglucose | 940.68 | SC160 | L-Norleucine | 131.17 |
| SC35 | Arctigenin | 372.41 | SC98 | 4-Hydroxyacetophenone | 136.15 | SC161 | Hernandezine | 652.77 |
| SC36 | Lanatoside C | 985.12 | SC99 | Lycorine (hydrochloride) | 323.77 | SC162 | Hanfangichin B (impurified) | 608.74 |
| SC37 | Cephalotaxlen | 315.36 | SC100 | Tizoxanide | 265.25 | SC163 | Ouabain | 584.67 |
| SC38 | Curcumin | 368.38 | SC101 | Valproic acid (sodium salt) | 166.19 | SC164 | N,N'-(hexane-1,6-diyl)bis(2-(2,7-bis(2-(diethylamino)ethoxy)-9H-fluoren-9-ylidene)hydrazine-1-carboxamide) | 1017.38 |
| SC39 | Camphor | 152.23 | SC102 | Efavirenz | 315.68 | SC165 | 2-(2,7-bis(2-morpholinoethoxy)-9H-fluoren-9-ylidene)hydrazine-1-carbothioamide | 511.65 |
| SC40 | Glycyrrhizic acid | 822.93 | SC103 | Betulinic acid | 456.70 | SC166 | 4-((3R,5S,8R,9S,10R,11R,13R,14S,17S)-5,11,14-trihydroxy-10-(hydroxymethyl)-13-methyl-3-(((2S,3S,4R,5R,6S)-3,4,5-trihydroxy-6-methyltetrahydro-2H-pyran-2-yl)oxy)hexadecahydro-1H-cyclopenta[a]phenanthren-17-yl)furan-2(5H)-one | 568.67 |
| SC41 | Dehydroandrographolide succinate | 532.58 | SC104 | Trilobatin | 436.41 | SC167 | 5-chloro-N-(2,6-dichloro-4-nitrophenyl)-2-hydroxybenzamide | 361.57 |
| SC42 | Isomangiferin | 422.34 | SC105 | 4,5-Dicaffeoylquinic acid | 516.45 | SC168 | VE607 | 465.46 |
| SC43 | Emodin | 270.24 | SC106 | Scutellarein | 286.24 | SC169 | SSAA09E3 | 327.34 |
| SC44 | Isoliquiritigenin | 256.25 | SC107 | Valproic acid | 144.21 | SC170 | (2R,2'R)-3,3'-(1,4-phenylenebis(oxy))bis(1-(piperidin-1-yl)propan-2-ol) | 392.54 |
| SC45 | Kaempferol | 286.24 | SC108 | Artemisinin | 282.33 | SC171 | 3,3'-(1,3-phenylenebis(oxy))bis(1-(4-methylpiperidin-1-yl)propan-2-ol) | 420.6 |
| SC46 | Psoralen | 186.16 | SC109 | Raltegravir (potassium salt) | 482.51 | SC172 | 2-(1-(5-chlorothiophen-2-yl)ethylidene)hydrazine-1-carbothioamide | 233.74 |
| SC47 | 9-Aminoacridine | 194.23 | SC110 | Mizoribine | 259.22 | SC173 | N-(4-(4-methylpiperazin-1-yl)benzyl)-4-(pyrrolidin-1-ylmethyl)benzamide | 392.55 |
| SC48 | β-Cyclodextrin | 1134.98 | SC111 | Theaflavin | 564.49 | SC174 | YM-201636 | 467.48 |
| SC49 | Sophocarpine | 246.35 | SC112 | Gramine | 174.24 | SC175 | Amiodarone hydrochloride | 681.77 |
| SC50 | Geldanamycin | 560.64 | SC113 | Artesunate | 384.42 | SC176 | APY0201 | 413.48 |
| SC51 | Xanthone | 196.20 | SC114 | Bevirimat | 584.83 | SC177 | Salinomycin sodium salt | 772.98 |
| SC52 | alpha-Mangostin | 410.46 | SC115 | Elemicin | 208.25 | SC178 | Dronedarone Hydrochloride | 593.22 |
| SC53 | Oroxylin A | 284.26 | SC116 | Fangchinoline | 608.72 | SC179 | Verapamil hydrochloride | 491.06 |
| SC54 | Oxyresveratrol | 244.24 | SC117 | Erythromycin Ethylsuccinate | 862.05 | SC180 | Fangchinoline | 608.71 |
| SC55 | Honokiol | 266.33 | SC118 | Isoferulic acid | 194.18 | SC181 | Isofangchinoline | 608.71 |
| SC56 | α-Lipoic Acid | 206.33 | SC119 | Chebulagic acid | 954.66 | SC182 | Tetrandrine | 622.75 |
| SC57 | Lapachol | 242.27 | SC120 | 4'-O-Methylbavachalcone | 338.40 | SC183 | Berbamine hydrochloride | 681.65 |
| SC58 | Adenosine 5'-monophosphate monohydrate | 365.24 | SC121 | Spermine | 202.34 | SC184 | Dauricine | 624.76 |
| SC59 | Phillyrin | 534.55 | SC122 | Pseudolaric Acid B | 432.46 | SC185 | Neferine | 624.77 |
| SC60 | Hypericin | 504.44 | SC123 | Oxytetracycline | 460.43 | SC186 | Liensinine | 610.74 |
| SC61 | Desaminotyrosine | 166.18 | SC124 | Baicalein | 270.24 | SC187 | Daurisoline | 610.74 |
| SC62 | (-)-Epigallocatechin Gallate | 458.37 | SC125 | Catechin | 290.27 | SC188 | Isoliensinine | 610.75 |
| SC63 | Anthraquinone | 208.21 | SC126 | Cytarabine | 243.22 |  |  |  |

**Table S2**

**Supplementary Tab. 2** The inhibitory activity of 19 hit compounds and aloxistatin against SARS-CoV-2 S-G614 and VSV-G pseudoviruses.

| **Compounds** | **293T-ACE2** | **S-G614 Pseudovirus** | **SI (cytotoxicity)** | **VSV-G Pseudovirus** | **SI (specificity)** |
| --- | --- | --- | --- | --- | --- |
|  | **CC_50_(μM)** | **EC_50_(μM)** |  | **EC_50_(μM)** |  |
| SC8 | ＞50 | 14.58 | ＞3.43 | ﹣ | ﹣ |
| SC9 | ＞50 | 0.32 | ＞158.58 | ＞50 | ＞158.58 |
| SC16 | ＞50 | 34.60 | ＞1.45 | **﹣** | **﹣** |
| SC24 | 33.5 | 15.66 | 0.90 | **﹣** | **﹣** |
| SC33 | ＞50 | 10.12 | ＞4.94 | ＞50 | ＞4.94 |
| SC96 | ＞50 | 0.28 | ＞176.87 | 10.84 | 38.34 |
| SC102 | ＞50 | 0.00038 | ＞130890 | 0.000723 | 1.89 |
| SC109 | ＞50 | 0.00020 | ＞247647 | 0.0359 | 177.86 |
| SC161 | ＞50 | 0.11 | ＞448.83 | ＞50 | ＞448.83 |
| SC171 | ＞50 | 3.83 | ＞13.06 | ＞50 | ＞13.06 |
| SC180 | ＞50 | 0.94 | ＞53.39 | 4.39 | 4.69 |
| SC181 | ＞50 | 1.11 | ＞45.00 | 3.95 | 3.56 |
| SC182 | ＞50 | 0.90 | ＞55.80 | 27.87 | 31.10 |
| SC183 | ＞50 | 1.28 | ＞38.94 | ＞50 | ＞38.94 |
| SC184 | ＞50 | 1.43 | ＞34.99 | ＞50 | ＞34.99 |
| SC185 | ＞50 | 0.95 | ＞52.87 | ＞50 | ＞52.87 |
| SC186 | ＞50 | 1.00 | ＞49.80 | ＞50 | ＞49.80 |
| SC187 | ＞50 | 2.48 | ＞20.13 | ＞50 | ＞20.13 |
| SC188 | ＞50 | 1.29 | ＞38.88 | 7.02 | 5.45 |
| Aloxistatin | ＞50 | 0.923 | ＞54.17 | ＞50 | ＞54.17 |
| The data represent results from three separate experiments | | | | | |

**Materials and Methods**

**Plasmids**

The codon-optimized gene encoding SARS-CoV-2 spike (S) protein (GenBank: QHD43416) with 19 amino acids deletion at the C-terminal was synthesized by Sino Biological Inc (Beijing, China), and cloned it into the pCMV3 vector between the restriction enzyme *Kpn*I and *Xba*I sites (denoted as pS-D614). The recombinant plasmid pS-D614 was used as template, and the D614G mutant S-expressing plasmid (denoted as pS-G614) was constructed by site-directed mutagenesis. N501Y.V1 (Variant 1) mutant Spike proteins of SARS-CoV-2 were codon-optimized and synthesized by GenScript Inc (Nanjing, China) and cloned into pCMV3 vector (denoted as pS-Variant1). N501Y.V2 (Variant 2) mutant Spike-expressing plasmid (denoted as pS-Variant2) was constructed by site-directed mutagenesis, with pS-D614 plasmid as a template. SARS-CoV S-expressing plasmid (Cat: VG40150-ACGLN, named as pS-SARS) and MERS-CoV S-expressing plasmid (Cat: VG40069-CF, named as pS-MERS) were obtained from Sino Biological Inc (Beijing, China). The VSV-G-expressing plasmid pMD2.G was donated by Prof. Ding Xue from Tsinghua University (Beijing, China). The HIV-1 NL4-3 ΔEnv Vpr luciferase reporter vector (pNL4-3.Luc.R-E-) constructed by N. Landau was donated by Prof. Cheguo Cai from Wuhan University (Wuhan, China). Human ACE2 and DPP4 expression plasmids were derived from GeneCopoeia (Guangzhou, China).

**Cell lines and cell culture**

HEK 293T, A549, and Calu3 cells were purchased from the American Type Culture Collection (ATCC, Manassas, VA, USA). Cells were cultured at 37 ºC and 5% CO_2_ atmosphere in Dulbecco’s modified Eagle medium (DMEM; Hyclone, Waltham, MA, USA) containing 10% fetal bovine serum (FBS; Gibco, Rockville, MD, USA), 100 mg/mL of streptomycin, and 100 units/mL of penicillin. HEK 293T cells transfected with human ACE2 and DPP4 (293T-ACE2, 293T-DPP4) were cultured under the same conditions with the addition of G418 (0.5 mg/mL) to the medium.

**Antigens and antibodies**

The RBD domain of SARS-CoV-2 S protein (His-tag) were synthesized by Prof. Xuefei Cai at Key Laboratory of Molecular Biology for Infectious Diseases (Ministry of Education), Chongqing Medical University. The anti-RBD monoclonal antibody was kindly provided by Prof. Aishun Jin from Chongqing Medical University. RBD-binding peptide SBP1 derived from human ACE2 α helix 1 (Ac-IEEQAKTFLDKFNHEAEDLFYQS-NH_2_) was synthesized by GenScript (Nanjing, China).

**Compounds and reagents**

Custom compound library containing 188 small molecules, remdesivir, and aloxistatin (E-64d) were purchased from MedChemExpress (HY-L027) and Chemdiv. BAPTA-AM (T6245) was purchased from TargetMol. All the compounds were dissolved in dimethyl sulfoxide (DMSO) at a stock concentration of 20 mM.

**Cell cytotoxicity assay**

The CellTiter 96® AQ_ueous_ One Solution Cell Proliferation Assay (G3582, Promega, USA) was used to assess cell viability according to the product’s description. Briefly, HEK 293T cells were dispensed into 96-well plate (2x10^4^ cells/well), cultured in medium containing gradient concentrations of the compound for 72 hours at 37 ºC in a humidified 5% CO_2_ incubator. The cells were incubated with 100 µl fresh medium after removal of the medium. Then 20 µl of CellTiter 96® AQueous One Solution Reagent was added into each sample well and incubating the plate was incubated at 37°C for 1-4 hours in a humidified 5% CO_2_ atmosphere. The absorbance at 490 nm was measured using a microplate reader (Synergy H1, BioTek, USA).

**Pseudovirus production and quantification**

Pseudotyped viruses were produced as previously described^1^. Briefly, 5×10^6^ HEK 293T cells were co-transfected with 6 μg each of pNL4-3.Luc.R-E- and recombinant plasmid (pS-SARS, pS-MERS, pS-D614, pS-G614, pS-Variant1, or pS-Variant2) using Lipofectamine 3000 Transfection Reagent (Invitrogen, Rockville, MD) according to the manufacturer’s instructions. After 48 h transfection, pseudotyped viruses expressing S-SARS, S-MERS, S-D614, S-G614, N501Y.V1, and N501Y.V2 spike protein were harvested, centrifuged and filtered through 0.45 μm filters, and subsequently stored at -80°C. 293T cells were co-transfected with pNL4-3.Luc.R-E- and pMD2.G plasmid to collect the VSV-G pseudovirus.

The copies of the pseudovirus were expressed as numbers of viral RNA genomes per mL of viral stock solution and determined using RT-qPCR with primers and a probe that targeting LTR. Sense primer: 5′-TGTGTGCCCGTCTGTTGTGT-3′, anti-sense primer: 5′-GAGTCCTGCGTCGAGAGAGC-3′, probe: 5′-FAM-CAGTGGCGCCCGAACAGGGA-BHQ1-3′. Briefly, viral RNAs were extracted according to the manufacturer’s instructions with TRIzol reagent (Invitrogen). Then, the TaqMan One-Step RT-PCR Master Mix Reagents (Applied Biosystems, Thermo Fisher) was used to amplify total RNAs. pNL4-3.Luc.R-E- vector with certain copies was used to generate standard curves. All the pseudotyped viruses were titrated to the uniform titer (copies/mL) for the following research.

**Compound screening**

For pseudovirus-based inhibition assay, 188 compounds were screened via luciferase activity. The lentivirus-based pseudotype system was generated in the envelope-defective HIV-1 backbone, so VSV-G pseudovirus was used as a control to exclude compounds that have anti-HIV-1 activities. HEK 293T cells cultured in 96-well plate (2x10^4^ cells/well) were incubated with each compound (20 μM) for 1 hour and were infected with the same amount of pseudovirus (3.8 × 10^4^ copies in 50 μL). The cells were replaced to fresh DMEM medium 8 h post-infection. Cells were lysed by 30 μl lysis buffer (Promega, Madison, WI, USA) at 72 h post-infection to measure RLU with luciferase assay reagent (Promega, Madison, WI, USA) according to the product description. All data were performed at least three times and expressed as means ± standard deviations (SDs).

**Cell-cell fusion assay**

HEK 293T effector cells were transfected with plasmid pAdTrack-TO4-GFP encoding green fluorescent protein (GFP) or pS-G614 encoding the corresponding SARS-CoV-2 S protein. 293T-ACE2 cells were used as target cells. At 8 h post-transfection, the effector cells were washed twice with PBS and were pretreated with compounds or DMSO as control for another 16h. Subsequently, the effector cells were overlaid on target cells at a ratio of approximately one S-expressing cell to two receptor-expressing cells with about 90% confluent. After a 4-hour coculture, images of syncytia were captured with an inverted fluorescence microscope (Nikon eclipse Ti, Melville, NY).

For quantification of cell-cell fusion, three fields were randomly selected in each well to count the fused and unfused cells. The fused cells were at least twice as large as the unfused cells, and the fluorescence intensity was weaker in fused cells since GFP diffusion from one effector cell to target cells. The percentage of cell-cell fusion was calculated as: [(number of the fused cells/number of the total cells) × 100%].

**Competitive ELISA**

The recombinant RBD proteins derived from SARS-CoV-2 were coated on 96-well microtiter plate (50ng/well) at 4°C overnight. After blocked with blocking buffer (5% FBS and 2% BSA in PBS) for 1 hour at 37°C, serial dilution solutions of compounds, ACE2 peptide SBP1 or DMSO were added into the plates and incubated at 37°C for 1 hour. Plates were washed five times with phosphate-buffered saline, 0.05% Tween-20 (PBST) to remove the free drug or DMSO. The wells were incubated with mouse anti-RBD monoclonal antibody (1:1000 dilution) for 1 hour at 37°C, and then washed with PBST five times and incubated with Horseradish peroxidase (HRP)-conjugated goat anti-mouse antibody (Abmart, Shanghai, China) for 1 hour at 37°C. TMB substrate was added and incubated for 15 minutes at 37°C for color development, finally the absorbance at 450 nm was measured by a microplate reader.

**Differential scanning fluorimetry**

Briefly, purified His-RBD was diluted to 200 μg/mL in PBS buffer containing Sypro Orange 5× (ThermoFisher) in a 96-well white PCR plate. Compounds and ACE2 derived peptide SBP1 were added at a final concentration of 20 μM. All samples were tested in triplicate with a Bio-Rad RT-PCR system. The samples were first equilibrated at 25 °C for 3 min, then heated from 25 °C to 85 °C with a step of 1 °C per 1 min, and the fluorescence signals were continuously collected using CFX Maestro. T_m_ values were calculated as previously described^2^.

**Functional analysis of Ca^2+^ in pseudovirus infectivity**

293T-ACE2 cells were seeded in 96-well plate (2x10^4^ cells/well) and incubated at 37 °C for 8h. For extracellular calcium depletion assays, cells were washed three times using PBS with or without Ca^2+^. Calcium-free medium with or without 2 mM calcium chloride and 5 μM compounds were added and incubated for 1h at 37°C. Next, S-G614 pseudovirus was added to the cells for 8 h at 37°C. For intracellular calcium depletion assays, cells were washed three times using PBS with or without Ca^2+^, DMEM with BAPTA-AM (20 μM) or DMSO and gradient concentrations of compounds were added and incubated for 2 h at 37°C. In the following, S-G614 pseudovirus was added to infect the cells for 8 h at 37°C. For both types of assays, complete medium was then added after removed the medium. The cells were measured by luciferase activity.

**Cellular cholesterol measurement**

Cellular cholesterol was measured using the Cholesterol/Cholesterol Ester-Glo™ Assay (J3190, Promega, USA) according to the product’s description. In short, HEK 293T-ACE2 cells were assigned to 96-well plates (4x10^4^ cells/well) and cultured for 24 hours at 37 °C with 5 μM compounds. DMSO was used as negative control. The medium in the 96-well plate was removed and cells was washed twice with 200 µl PBS. Then, 50 µl of cholesterol lysis solution was added, shaking the plate carefully and incubating for 30 minutes at 37°C. Following, 50 µl of cholesterol detection reagent was added with esterase or without esterase to all wells and the plate was incubated at room temperature for 1 hour. Finally, recording luminescence with a plate-reading luminometer. Total cholesterol concentration was calculated by comparing the luminescence of samples and controls under the same condition.

**Cytopathic effect (CPE) assay and quantification of SARS-CoV-2 infection**

Vero E6 cells were dispensed into 96-well plate (4.0x10^4^ cells/well), pre-treated with medium containing compounds or DMSO for 1 hour at 37 ºC. Then 60 μl DMEM medium which supplemented with compounds or DMSO was replaced immediately, the same amount of SARS-CoV-2 (100 TCID_50_/well) was added and incubated for 1 hour at 37 ºC. The mixture was removed and the cells were washed twice with PBS, and cultured with 100 μl fresh medium for 48 hours at 37 ºC with 5% CO_2_ atmosphere. Cytopathic effects (CPE) induced by the virus was observed using microscope at 48 h post-inoculation. Cell culture supernatant was collected at 48 h post-infection for viral RNA quantification using the novel coronavirus real-time RT-PCR Kit (Shanghai ZJ Bio-Tech Co, Ltd, Shanghai, China). Remdesivir (5 μM) was used as positive control in the experiment.

**Statistical analyses**

Data were analyzed using GraphPad Prism version 6.0 software and were presented as means ± SD. Statistical significance was determined using ANOVA for multiple comparisons. Student’s t test was applied to compare the two groups. Differences with P values < 0.05 were deemed statistically significant.

**Data availability**

Data supporting the results of this study can be obtained from the author upon request

**Reference**

1 Hu, J. *et al.* Development of cell-based pseudovirus entry assay to identify potential viral entry inhibitors and neutralizing antibodies against SARS-CoV-2. *Genes Dis* (2020).

2 Niesen, F. H., Berglund, H. & Vedadi, M. The use of differential scanning fluorimetry to detect ligand interactions that promote protein stability. *Nat Protoc* **2**, 2212-2221 (2007).
